# The Impact of Antenatal Depression on Perinatal Outcomes in Australian Women

**DOI:** 10.1371/journal.pone.0169907

**Published:** 2017-01-17

**Authors:** John Eastwood, Felix A. Ogbo, Alexandra Hendry, Justine Noble, Andrew Page, for the Early Years Research Group (EYRG)

**Affiliations:** 1 School of Women’s and Children’s Health, The University of New South Wales, Kensington, Sydney, NSW, Australia; 2 Menzies Centre for Health Policy, Charles Perkins Centre, School of Public Health, Sydney University, Sydney NSW, Australia; 3 School of Public Health, Griffith University, Gold Coast, QLD, Australia; 4 Department of Community Paediatrics, Sydney Local Health District, Croydon Community Health Centre, Croydon NSW Australia; 5 Ingham Institute for Applied Medical Research, Liverpool NSW Australia; 6 Centre for Health Research, School of Medicine, Western Sydney University, Campbelltown Campus, Locked Bag 1797, Penrith NSW Australia; 7 Coverage and Surveillance, National Centre for Immunisation Research and Surveillance, The Children’s Hospital at Westmead, Locked Bag 4001, Westmead NSW, Australia; Royal Children's Hospital, AUSTRALIA

## Abstract

**Background:**

In Australia, there is limited evidence on the impact of antenatal depression on perinatal outcomes. This study investigates the association between maternal depressive symptoms during pregnancy and key perinatal outcomes, including birth weight, gestational age at birth, breastfeeding indicators and postnatal depressive symptoms.

**Method:**

A retrospective cohort of mothers (N = 17,564) of all infants born in public health facilities within South Western Sydney Local Health District and Sydney Local Health District in 2014, in the state of New South Wales (NSW), Australia, was enumerated from routinely collected antenatal data to investigate the risk of adverse perinatal outcomes associated with maternal depressive symptoms during pregnancy. Antenatal depressive symptoms were measured using the Edinburgh Postnatal Depression Scale (EPDS). Logistic regression models that adjusted for confounders were conducted to determine associations between antenatal depressive symptoms and low birth weight, early gestational age at birth (<37 weeks), breast feeding indicators and postnatal depressive symptoms.

**Results:**

The prevalence of maternal depressive symptoms during pregnancy was 7.0% in the cohort, and was significantly associated with postnatal depressive symptoms [Adjusted Odd Ratios (AOR) = 6.4, 95% CI: 4.8–8.7, P<0.001]. Antenatal depressive symptoms was associated with a higher odds of low birth weight [AOR = 1.7, 95% CI: 1.2–2.3, P = 0.003] and a gestational age at birth of <37 weeks [AOR = 1.3, 95% CI: 1.1–1.7, P = 0.018] compared to women who reported lower EPDS scores in antenatal period. Antenatal depressive symptoms were not strongly associated with non-exclusive breast feeding in the early postnatal period.

**Conclusion:**

Maternal depressive symptoms in the antenatal period are strongly associated with postnatal depressive symptoms and adverse perinatal outcomes in Australian infants. Early identification of antenatal and postnatal depressive symptoms, and referral for appropriate management could benefit not only the mother’s mental health, but also the infant’s health and development.

## Introduction

Globally, depression is a significant source of disease burden among females, and has been ranked by the Global Burden of Disease Study (2015) as the leading source of disease burden in Australian women in terms of disability-adjusted life years [1]. Depression during pregnancy has been shown to be prevalent among women (ranging from 3.0 to 22.6%) [2, 3], and is also associated with considerable distress, lost productivity and poorer maternal mental health behaviours [4, 5]. Antenatal depression can also predispose mothers to higher rates of depression later in life [6]. Previous studies have reported that antenatal depression was one of the strongest determinants (in terms of effect size) of postnatal depression (PND) [7].

Studies from the United Kingdom [8], the United States [9], Nigeria [10], Pakistan [11] and Brazil [9, 12], including recent systematic reviews [13, 14] have reported that maternal depression during pregnancy is associated with poor perinatal outcomes such as low birth weight, growth retardation, diarrhoea episodes and sub-optimal infant feeding patterns. Poor immunisation rates, frequent hospitalisation and higher health expenses have also been reported in children of depressed mothers [15–17].

Additionally, early childhood psychological problems arising from maternal depression during pregnancy are also associated with subsequent psychiatric disorders, school absence, and poor academic performance and social functioning in adolescences and adults [18–20]. Documented mechanisms of the effect of depression include negative health behaviours from depression, such as substance use and poor nutrition [21], dysregulation of the hypothalamic-pituitary-adrenal axis [22] and increased inflammation [23, 24] which affects fetal biological milieu; ongoing stress and poor social functioning.

Despite prior evidence on the impact of antenatal depression on perinatal outcomes in other contexts, previous literature reviews have observed ‘variation and inconclusive’ evidence regarding the associations between depression during pregnancy and adverse perinatal outcomes [25–30]. These variations in the evidence may be due to a range of factors including, differences in methodology and assessment of depression, sample size, and inconsistency in adjustment for potential confounders. The authors therefore, suggested that further research on the link between prenatal depression and perinatal outcomes are needed.

In Australia, the broader impact of perinatal depression is under-researched [31]. Policy recommendations issued by the *Beyondblue* National Postnatal Depression Program (2001–2005) have recognised the need for continuing research in perinatal depression. Context-specific evidence is also needed to determine the extent to which mothers with antenatal depression continue to be symptomatic postnatally, in addition to the impact of maternal depression during pregnancy on the infant. Few studies on antenatal depression have been conducted in the Australian context using a validated screening tool such as the Edinburgh Postnatal Depression Scale (EPDS) [32–36]. However, these studies were not focused on the impact of antenatal depression on perinatal outcomes, which is the main aim of this study.

This is the first Australian study to use routinely collected health service data to investigate the impact of antenatal depression on perinatal outcomes and to consider how antenatal factors may modify the risk of adverse perinatal outcomes. Additionally, this study provides information on antenatal depression and perinatal outcomes among a diverse Australian population in New South Wales. Specifically, this study investigates the association between maternal depressive symptoms during pregnancy and key perinatal outcomes, including (i) birth weight, (ii) gestational age at birth, (iii) breast feeding indicators, and (iv) postnatal depressive symptoms.

## Methods

### Data source

The data used for this study are similar to those used for the New South Wales (NSW) Perinatal Data Collection (PDC), which is a population-based surveillance system covering all births in NSW public and private hospitals, as well as home births. Demographic data and information on maternal health, the pregnancy, labour, birth, and perinatal outcomes are collected to inform the development of policy and intervention aimed at improving the health of mothers and newborns in NSW. A detailed description of the data is provided elsewhere [37, 38].

For this study, a retrospective cohort of mothers (N = 17,564) of all infants born in public health facilities within the South Western Sydney Local Health District (SWSLHD) and the Sydney Local Health District (SLHD) in 2014, in the state of New South Wales (NSW), Australia, was established using routinely collected antenatal data obtained from the relevant Local Health District electronic medical records. Antenatal data (collected by qualified midwives) were linked using individual identifiers to routinely collected postnatal data relating to perinatal outcomes and maternal health outcomes. The mean age of women in this cohort was 31 years (SD = 5.5, range 14–54 years). A high proportion of women (53%) had a country of birth other than Australia, predominantly Middle Eastern countries (10%), South East Asia (8%) and Southern Asia (8%), with the cohort comprising women from over 25 nationalities. Approximately 2% of the study population were Indigenous Australians.

### Study setting

The geographic area of these health districts captures approximately 51.9% of the Sydney metropolitan region, and represents a population catchment of more than 1,457,100 people, with a diverse multicultural and linguistic background [39, 40]. The SWSLHD is located in the west of Sydney and consist of Local Government Areas of Bankstown, Fairfield, Liverpool, Campbelltown, Camden, Wollondilly and Wingecarribee, with majority of the population born overseas (35.8%) compared to NSW (25.7%) [41]. The SLHD is located in the centre and inner west of Sydney, and comprises the Local Government Areas of Leichhardt, Marrickville, Canterbury, Canada Bay, City of Sydney (part), Ashfield, Burwood and Strathfield, with almost half (49%) of the population born overseas [42]. In this setting, antenatal care (ANC) services are provided in the hospital and non-hospital environment. The hospital settings include doctor’s clinics, midwives clinics or birth centres. In the non-hospital sites, ANC services are provided by the general practitioner (GP) as part of the GP Shared Care program or by private obstetricians. The proportion of women attending ANC services in this areas within the first trimester (1–13 weeks) is high (66.4%) [38].

Additionally, these health districts provide services to some of the most socio-economically disadvantaged areas in the Sydney metropolitan region. The Australia health care system is a comprehensive network of both public and private provider, and supporting mechanisms. While the Australian federal government provides the universal health care system through Medicare, the state and territory governments administer basic elements of health care within their respective jurisdictions, such as the management of hospitals [43].

### Ethics

Institutional ethics approvals were obtained from the South Western Sydney Local Health District and the Sydney Local Health District Ethics committees to conduct this data linkage study. Data used for this project were anonymous and no individuals were contacted (Approval numbers HREC: LNR/11/LPOOL/463; SSA: LNRSSA/11/LPOOL/464 & Project No: 11/276 LNR; Protocol No X12-0164 & LNR/12/RPAH/266). The data used for the analysis are accessed in accordance with ethical protocols that only allow unit record information to be released to investigators included in the ethics committee submission for study approvals.

### Maternal depression

Maternal depressive symptoms during pregnancy were based on the Edinburgh Postnatal Depression Scale (EPDS) completed at first antenatal care visit of mothers by qualified midwives. Given the multicultural and linguistically diverse context of the area, the EPDS was administered to non-English speaking mothers through qualified interpreters. The EPDS has been translated and validated in a number of non-English speaking contexts [44], including studies of Iranian [45, 46], Bangladeshi [47, 48], Chinese [49], Serbian [50], and Greek women [51]. This population is part of the multi-cultural community in the study cohort. Maternal depressive symptoms based on the EPDS were also collected at a postnatal visit within the first six weeks and were used as a measure of postnatal depressive symptoms. At both time points, the total number of depressive symptoms was tallied to obtain a total score (out of 30), which was then coded as a categorical variable (score ≥13 or score <13) to indicate scores that are likely to suggest depressive disorder [52]. In these local health districts, a woman who reported a higher EPDS score of ≥13 is referred to the psychiatric clinician for formal assessment of depression and appropriate management.

The EPDS rates the severity of depressive symptoms experienced over the previous 7 days. Five of the items explore dysphoric mood, two explore anxiety, and three assess guilt and suicidal thoughts. Maternal depressive symptoms during pregnancy was the main exposure for the present study, with postnatal maternal depressive symptoms one of the outcomes of interest. Globally, the EPDS is the most widely accepted screening tool in the perinatal period, with a reported sensitivity of 68–86%, and specificity of 78–96% [52, 53]. In an Australian sample of 4,148 women, the reported sensitivity was 100% and specificity was 89% [54]. The EPDS has also been validated and recommended for use in Australia by the *Beyondblue* National Postnatal Depression Program [31]. An EPDS score of ≥12 showed a positive predictive value (PPV) for clinical depression [53], with a consistent PPV of approximately 70% in a number of studies [52, 54, 55]. The EPDS tool has also been validated for use antenatally [56].

### Perinatal outcomes

Perinatal outcomes of interest were birth weight and gestational age at birth. Birth weight was categorised as a binary variable, as low birth weight (<2,500 grams) and normal birth weight (≥2,500 grams). Gestational age (GA) at birth was also categorised as a binary variable, defined as preterm birth (<37 weeks) or full term birth (≥37 weeks). Gestational age is the duration of pregnancy in completed weeks from the first day of the last normal menstrual period. Gestational age was measured from ultrasound scan measurement, where a woman has an unsure date of last normal menstrual period.

### Breastfeeding indicators

Breastfeeding indicators of interest included early initiation of breastfeeding at delivery, exclusive breastfeeding at delivery, at discharge and exclusive breastfeeding at first postnatal visit within the first six weeks. Exclusive breastfeeding was defined as infants aged 0–5 months who received only breast milk (including expressed milk), but allowed oral rehydration solution, syrups of vitamins/medicines. These indicators were based on the World Health Organisation definitions for assessing infant and young child feeding practices [57]. In the analyses, non-exclusive breastfeeding was assessed as infants aged 0–5 months who received other liquids such as infant formula, fruit juice, water and water-based drinks, assessed at delivery, discharge and at six weeks postnatal.

### Confounding factors

A series of confounding factors were also considered in the present study based on previous studies [32, 34, 58, 59]. These factors include: maternal age, socio-economic status, Indigenous status, history of intimate partner violence, language spoken at home, body mass index (BMI), socio-economic status and history of antenatal health problems (such as diabetes mellitus and hypertension). Socio-economic status was based on the Socio-Economic Index for Areas, an area measure based on mother’s address provided [60]. Deciles of socio-economic status were categorised into High (top 10% of the population), Middle (middle 80% of the population) and Low (bottom 10% of the population) groups. Additionally, other factors previously associated with birth outcomes included alcohol consumption during pregnancy, smoking during pregnancy intervention received during delivery (i.e., instrumental or caesarean delivery). These variables were considered intermediaries between the main exposure (antenatal depression) and study outcomes (and were included in descriptive analyses and multiple imputation of missing data), and as such were not adjusted for in logistic regression models (as described below).

### Analytic strategy

The likelihood of adverse perinatal outcomes associated with maternal depressive symptoms during pregnancy was investigated in logistic regression models to determine if antenatal depressive symptoms were associated with (i) postnatal depressive symptoms, (ii) low birth weight, (iii) early gestational age at birth (<37 weeks), and (iv) breast feeding practices, including exclusive breastfeeding following delivery, at discharge, and the first postnatal visit within the first six weeks. Univariate models investigated the association between antenatal depressive symptoms and perinatal outcomes, followed by multivariate models incorporating the confounders described above. Univariate and multivariate linear regression models were also conducted with post-natal depressive symptom score specified as a (log transformed) continuous outcome variable, in a sensitivity analysis to investigate whether associations using a binary outcome may be a source of ascertainment bias due to the cut-point used to define cases and non-cases of post-natal depressive symptoms (as described above).

### Missing data

**Fig 1:**
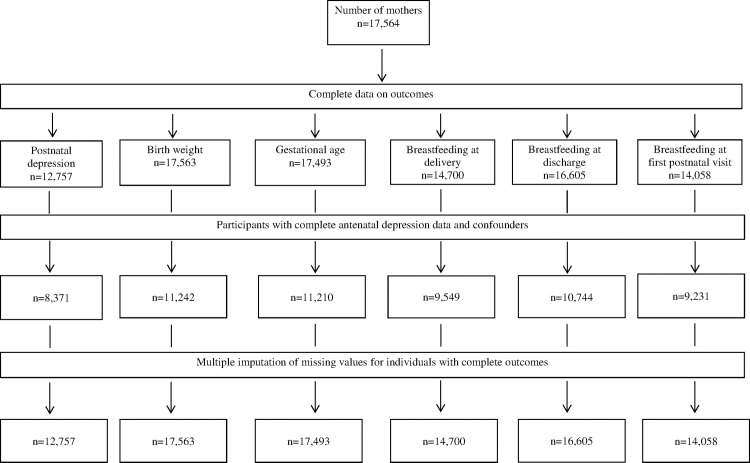
Flow chart of available data on postnatal depression, birth outcomes, and breast feeding behaviours of mothers of infants from South Western Sydney and Sydney Local Health Districts in 2014 (n = 17,564).

Sensitivity analyses were also conducted on an imputed dataset based on the original cohort comprising complete outcome data for each outcome of interest to examine the likely effect of missing data in study factors and confounders on observed odds ratio (Fig 1). Multivariate imputation by chained equations (MICE) [61] was used which assumes that data are Missing At Random (MAR) and that the known characteristics of participants can be used to estimate the characteristics of individuals with missing data, or who are lost to follow-up [62]. Multiple imputation was conducted using the *ice* command in Stata (Stata Corp, V.14.0, College Station, TX, USA) and based on 20 multiple imputations [63]. All outcome and study variables in the principal analysis were included in the multiple imputation modelling, as well as additional variables available on the dataset including BMI, Apgar score, hospital of birth, a history of child abuse, offspring sex, baby birth weight in grams, and gestational age in weeks. Revised odds ratio estimates from imputed data for comparison with the complete-case analysis were generated using the *mim* command.

## Results

The prevalence of maternal depressive symptoms during pregnancy was 7.0% in the cohort. The prevalence of low birth weight was 4.0% and a gestational age at birth less than 37 weeks was 10.9%. The prevalence of EPDS of 13 or more was 3.0% (Table 1).

**Table 1:** Prevalence of postnatal depressive symptoms and perinatal outcomes by study factor.

	Postnatal depressive symptoms			Low birth weight (<2500g)			Gestational age (<37 weeks)		
	Participants	Cases	%	Participants	Cases	%	Participants	Cases	%
**Total**	8,367	253	3.0	11,237	445	4.0	11,205	1,224	10.9
**Antenatal depressive symptoms**									
No	7,871	181	2.3	10,574	402	3.8	10,542	1,129	10.7
Yes	496	72	14.5	663	43	6.5	663	95	14.3
**Maternal age group**									
<20 years	404	10	2.5	552	15	2.7	551	65	11.8
20–39 years	7,816	236	3.0	10,483	417	4.0	10,454	1,131	10.8
>40 years	147	7	4.8	202	13	6.4	200	28	14.0
**SES category**									
High	1,003	135	4.0	4,822	202	4.2	1,314	530	11.0
Middle	3,965	99	2.5	5,100	200	3.9	5,088	561	11.0
Low	3,400	19	1.9	1,315	43	3.3	4,803	133	10.1
**Australian born**									
No	4,352	92	2.3	5,940	238	4.0	5,929	569	10.8
Yes	4,015	161	3.7	5,297	207	3.9	5,276	655	11.0
**Aboriginality**									
Non Indigenous	8,225	252	3.1	11,038	426	3.9	11,002	1,184	10.8
Indigenous	142	1	0.7	204	19	9.3	203	40	19.7
**BMI**									
Underweight	524	14	2.7	714	48	6.7	709	96	13.5
Normal weight	4,735	140	3.0	6,296	265	4.2	6,283	660	10.5
Overweight	1,867	66	3.5	2,523	77	3.1	2,519	282	11.2
Obese	1,241	33	2.7	1,704	55	3.2	1,694	186	11.0
**Antenatal problems**									
No	5,843	159	2.7	7,795	220	2.8	7,773	709	9.1
Yes	2,524	94	3.7	3,442	225	6.5	3,432	515	15.0
**Domestic violence**									
No	8,269	247	3.0	11,090	436	3.9	11,059	1,201	10.9
Yes	98	6	6.1	147	9	6.1	146	23	15.8
**Intervention at birth**									
No	7,357	213	2.9	9,874	313	3.2	9,844	964	9.8
Yes	1,010	40	4.0	1,363	132	9.7	1,361	260	19.1
**Alcohol consumption**									
No	8,226	250	3.0	11,048	440	4.0	11,017	1,209	11.0
Yes	141	3	2.1	189	5	2.6	188	15	8.0
**Smoking**									
No	7,736	235	3.0	10,283	363	3.5	10,255	1,092	10.6
Yes	631	18	2.9	954	82	8.6	950	132	13.9
**Language other than English spoken at home**									
No	6,491	173	2.7	8,601	340	4.0	8,574	950	11.1
Yes	1,876	80	4.3	2,636	105	4.0	2,631	274	10.4

The prevalence of non-exclusive breastfeeding following delivery was 10.5% and 11.4% at discharge, and was higher (16.5%) at the first post-natal visit (Table 2).

**Table 2:** Prevalence of breast feeding indicators by study factor.

	Non-exclusive breastfeeding at delivery			Non-exclusive breastfeeding at discharge			Non-exclusive breastfeeding at first postnatal visit		
	Participants	Cases	%	Participants	Cases	%	Participants	Cases	%
**Total**	9,545	1,007	10.5	10,739	1,228	11.4	9,227	1,503	16.3
**Antenatal depressive symptoms**									
No	9,025	941	10.4	10,120	1,146	11.3	8,690	1,399	16.1
Yes	520	66	12.7	619	82	13.2	537	104	19.4
**Maternal age group**									
<20 years	446	51	11.4	532	47	8.8	456	63	13.8
20–39 years	8,924	916	10.3	10,020	1,116	11.1	8,618	1,364	15.8
>40 years	175	40	22.9	187	65	34.8	153	76	49.7
**SES category**									
High	1,147	589	14.6	1,283	612	13.3	1,146	692	18.3
Middle	4,377	363	8.3	4,874	534	11.0	4,303	680	15.8
Low	4,021	55	4.8	4,582	82	6.4	3,778	131	11.4
**Australian born**									
No	4,975	510	11.2	5,696	805	16.0	4,904	998	23.1
Yes	4,570	497	10.0	5,043	423	7.4	4,323	505	10.3
**Aboriginality**									
Non Indigenous	9,374	970	10.3	10,552	1,173	11.1	9,064	1,434	15.8
Indigenous	171	37	21.6	187	55	29.4	163	69	42.3
**BMI**									
Underweight	620	66	10.6	675	73	10.8	578	85	14.7
Normal weight	5,390	474	8.8	6,063	548	9.0	5,271	688	13.0
Overweight	2,130	248	11.6	2,398	303	12.6	2,037	375	18.4
Obese	1,405	219	15.6	1,603	304	19.0	1,341	355	26.5
**Antenatal problems**									
No	6,647	717	10.8	7,481	867	11.6	6,381	1,069	16.7
Yes	2,898	290	10.0	3,258	361	11.1	2,846	434	15.2
**Domestic violence**									
No	9,432	985	10.4	10,606	1,198	11.3	9,117	1,475	16.2
Yes	113	22	19.5	133	30	22.6	110	28	25.5
**Intervention at birth**									
No	8,653	899	10.4	9,506	1,107	11.6	8,090	1,309	16.2
Yes	892	108	12.1	1,233	121	9.8	1,137	194	17.0
**Alcohol consumption**									
No	9,383	979	10.4	10,557	1,199	11.4	9,067	1,477	16.3
Yes	162	28	17.3	182	29	15.9	160	26	16.3
**Smoking**									
No	8,751	811	9.3	9,864	974	9.9	8,546	1,209	14.1
Yes	794	196	24.7	875	254	29.0	681	294	43.2
**Language other than English spoken at home**									
No	7,349	742	10.1	8,222	1,019	12.4	7,084	1,268	17.9
Yes	2,196	265	12.1	2,571	209	8.1	2,143	235	11.0

Maternal depressive symptoms during pregnancy was strongly associated with postnatal depressive symptoms (OR = 6.4, 95% CI 4.8–8.7, P<0.001) in the models that adjusted for confounders (Table 3). Similar strong associations were evident in adjusted models specifying postnatal depressive symptoms as a (log transformed) continuous outcome (*β* = 0.566 95%CI 0.493–0.639, P<0.001, not shown). Antenatal depressive symptoms was associated with a higher odds of low birth weight (OR = 1.7, 95%CI 1.2–2.3, P = 0.003) and a gestational age at birth of <37 weeks (OR = 1.3, 95%CI 1.0–1.7, P = 0.018), compared to women who reported lower EPDS scores in antenatal period. Antenatal depressive symptoms was associated with higher odds of non-exclusive breastfeeding following delivery, at discharge, and at the first postnatal visit in univariate analyses; however, these associations were attenuated following adjustment for confounders, with ORs ranging from 1.0 to 1.3 (Table 3). Findings from multiple imputation analyses were similar to the complete case analysis, suggesting that that missing information on confounders did not substantially affect findings.

**Table 3:** Associations between antenatal depressive symptoms and postnatal depressive symptoms, perinatal outcomes, and breast feeding behaviours of mothers of infants from South Western Sydney and Sydney Local Health Districts in 2014 (n = 17,564).

				Complete case				Multiple imputation			
Outcome	n	N	%	Unadjusted OR (95%CI) (a)	P value	Adjusted OR (95%CI) (b)	P value	Unadjusted OR (95%CI) (a)	P value	Adjusted OR (95%CI) (b)	P value
**Postnatal depressive symptoms**											
Antenatal depressive symptoms											
No	181	7,871	2.3	1.0		1.0		1.0		1.0	
Yes	72	496	14.5	7.2 (5.4–9.6)	<0.001	6.4 (4.8–8.7)	<0.001	7.3 (5.7–9.5)	<0.001	6.6 (5.1–8.7)	<0.001
**Low birth weight (<2500g)**											
Antenatal depressive symptoms											
No	402	10,574	3.8	1.0		1.0		1.0		1.0	
Yes	43	663	6.5	1.8 (1.3–2.4)	0.001	1.7 (1.2–2.3)	0.003	1.6 (1.2–2.2)	0.004	1.5 (1.1–2.1)	0.013
**Gestational age (<37 weeks)**											
Antenatal depressive symptoms											
No	1,129	10,542	10.7	1.0		1.0		1.0		1.0	
Yes	95	663	14.3	1.4 (1.1–1.8)	0.004	1.3 (1.1–1.7)	0.018	1.5 (1.2–1.8)	<0.001	1.3 (1.1–1.6)	0.004
**Non-exclusive breastfeeding at delivery**											
Antenatal depressive symptoms											
No	941	9,025	10.4	1.0		1.0		1.0		1.0	
Yes	66	520	12.7	1.3 (0.9–1.6)	0.103	1.1 (0.8–1.5)	0.454	1.3 (1.0–1.7)	0.031	1.1 (0.9–1.4)	0.371
**Non-exclusive breastfeeding at discharge**											
Antenatal depressive symptoms											
No	1,146	10,120	11.3	1.0		1.0		1.0		1.0	
Yes	82	619	13.2	1.2 (0.9–1.5)	0.145	1.2 (0.9–1.5)	0.167	1.4 (1.1–1.8)	0.002	1.4 (1.1–1.7)	0.010
**Non-exclusive breastfeeding at first post-natal visit.**											
Antenatal depressive symptoms											
No	1,399	8,690	16.1	1.0		1.0		1.0		1.0	
Yes	104	537	19.4	1.3 (1.0–1.6)	0.047	1.3 (1.1–1.7)	0.015	1.3 (1.1–1.6)	0.017	1.3 (1.0–1.6)	0.021

(a): Unadjusted Odds Ratio (OR)

(b): Adjusted for maternal age, socio-economic status, Indigenous status, history of intimate partner violence, language spoken at home, body mass index and history of antenatal problems (such as diabetes mellitus and hypertension).

## Discussion

The prevalence of maternal depressive symptoms during pregnancy was 7.0%, consistent with estimate reported by the *Beyondblue* National Postnatal Depression Program [31], indicating that the EPDS screening tool currently being used by the Local Health Districts may likely identify women with depressive symptoms from an early stage. Maternal depressive symptoms during pregnancy were significantly associated with postnatal depressive symptoms and poor perinatal outcomes. The association between maternal depressive symptoms during pregnancy and non-exclusive breastfeeding at delivery, discharge and first postnatal was attenuated after adjustment for confounders in the analyses.

A range of study limitations must be considered when interpreting the findings. The EPDS does not identify all women with depression, and some women with high scores will not be clinically depressed. The outcome and some of the exposures (e.g., non-EBF) as well as the EPDS were based on self-report which may lead to a recall and/or measurement bias that may either underestimate or overestimate the association between antenatal depressive symptoms and perinatal outcomes as well as postnatal depressive symptoms. Similarly, unmeasured confounding factors (such as maternal education, multi-parity or level of support services received prior to pregnancy) are also likely to affect the study findings. Additionally, although this study used an EPDS cut-off of ≥13 in the antenatal and postnatal periods; some studies have suggested that a score of 14 or 15 be used to diagnose minor depression during pregnancy and postnatal period [56, 64]. Data on antidepressant use were unavailable, information that may have significant impact on the outcome measures. Finally, the analyses were unable to separate women with a history of depression and/or postpartum depression from those with first-ever depressive symptoms during the postpartum period.

Despite these limitations, the study does have a number of strengths, suggesting that routinely collected antenatal data can provide important information on maternal-infant dyads in Sydney, NSW Australia. This study provides data on maternal depressive symptoms during pregnancy and perinatal outcomes among the most diverse women in the state of NSW, Australia, indicating that findings from this study may be extrapolated to other Australian states and territories with multi-cultural communities. The EPDS tool is internationally accepted and is employed in various facilities as a screening tool to identify women with symptoms suggestive of depression during pregnancy or postnatal period. The EPDS is recommended for use in the pregnancy period because it does not include somatic symptoms associated with depression that may produce incorrectly high scores in pregnancy as a result of somatic symptoms like lethargy. The study also investigated the potential bias due to missing data on study associations in a sensitivity analysis that imputed missing information on confounding variables, and was able to incorporate a range of potential confounding factors relating to socio-demographic and health service determinants of perinatal outcomes.

Even though in the current analysis, the prevalence of maternal depressive symptoms during pregnancy was not stratified by trimesters due to a lack of depression measures at visits subsequent to the first antenatal visit, previous studies have suggested that the prevalence of maternal depression during pregnancy can vary across trimesters [58, 65]. The highest prevalence of clinical depression has been reported in the first and third trimester of pregnancy [66, 67]. However, Bennet et al. (2004) found no statistical significance in the prevalence of maternal depressive symptoms throughout the different stages of pregnancy. Information on the peak periods of maternal depression during pregnancy may be useful for designing public health initiatives that specifically target women with early signs of mental disorders during pregnancy.

Consistent with previous reports [10, 65, 68], this study found an association between maternal depressive symptoms during pregnancy and adverse perinatal health outcomes even after adjusting for maternal health problems, suggesting that maternal depressive symptoms during pregnancy can lead to specific health issues early in life [13, 21]. Additionally, evidence from this study is consistent with previous findings which indicated that maternal depressive symptoms during pregnancy was significantly associated with postnatal depressive symptoms [69, 70]. Previous studies have reported an increase in the prevalence of postnatal depression among mothers who had maternal depression during pregnancy [58, 71]. In Australia, many health behaviours (such as optimal infant feeding practices) and health promotion measures (such as immunisation and adequate health-seeking behaviours) are largely initiated by the mother [72, 73], who is the primary care-giver in many households and communities. Accordingly, early detection and appropriate and timely treatment of antenatal and postnatal depressive symptoms is paramount to achieving optimal improvements in maternal and child health outcomes in Australia.

Maternal socio-economic status plays a key role in the onset of perinatal depression. In this study, statistical adjustments were made for a number of confounders (including maternal socio-economic status). A study from Italy found that low socio-economic status and multi-parity were associated with higher odds of developing depressive episode in the perinatal period [58]. A range of plausible reasons for why mothers from low socio-economic status develop depressive symptoms or clinical depression during the perinatal period have been suggested, including mother’s anxiety to care for the child because of limited resources, and additional tasks presented to a multiparous mother from the index child, particularly during the postpartum period [74].

This study found that the association between antenatal depressive symptoms and sub-optimal breastfeeding practices was weakened after adjustment for confounding factors. This finding is inconsistent with previous studies where antenatal and postnatal depressive symptoms was associated with sub-optimal breastfeeding practices [9, 75]. Furthermore, Rahman et al. (2004) from Pakistan reported that infants of prenatally depressed mothers were significantly more likely to have growth retardation and episodes of diarrhoea compared to the controls at all-time points [11], reflecting the adverse effect of sub-optimal infant practices. A study from the United Kingdom found that guilt was a major issue for depressed women who experienced breastfeeding difficulties, regardless of whether or not they continued breastfeeding [76]. Maternal depression has also been associated with thoughts and feelings of failing to be a ‘good mother’ [77].

Although low socio-economic status has been reported in previous studies as a risk factor for poor infant nutrition in Australia and the United States [78, 79]; antenatal and postnatal depression remain significant factors for poor infant feeding practices even after adjusting for socio-economic status [11]. Plausible mechanisms by which maternal depression may affect infant feeding practices include maternal emotional and physical issues, psychosocial factors (such as a lack of financial resources or limited family support), as well as reduced health seeking behaviours [11, 80]. Additionally, the impact of maternal depression during pregnancy on perinatal outcomes may also be due to the stimulation of the prenatal Hypothalamic Pituitary Adrenal (HPA) axis and epigenetic processes, where increase cortisol (an end product of HPA axis) has been documented [81, 82]. However, the full biological mechanisms through which antenatal depression can influence perinatal health outcomes are not yet fully understood [83, 84].

These findings have implications for policy makers, health administrators, health care professionals and the public. This study provides information on the impact of antenatal depressive symptoms on perinatal outcomes among multi-cultural Australian women using locally relevant data, where maternal depression during pregnancy may negatively influence mother-infant health outcomes. Efforts to reduce the impact of antenatal depression should be community-specific, and must consider these priority areas: community awareness and destigmatisation, family participation, prevention and early intervention, support of the primary health care system and targeted research [85]. Intervention studies that evaluate current and previous policy initiatives and the broader impacts of health system strengthening on antenatal depression and perinatal outcomes should be a key priority. Additionally, studies investigating important pre-pregnancy risk factors (such as smoking, intimate partner violence, partner support or alcohol use) associated with antenatal and postnatal depression in the Australian context are also warranted.

## Conclusion

Antenatal depressive symptoms were associated with maternal depressive symptoms during the postnatal periods and higher odds of adverse perinatal outcomes in a sample of Australian infants. Early identification of likely depressive symptoms using the EPDS, and subsequent referral for formal antenatal or postnatal risk assessment could benefit not only maternal mental health, but also the physical health, growth and development of the infant.

### Members of the Early Years Research Group

Amit Arora, Anne McKenzie, Bin Jalaludin, Elaine Tennant, Erin Miller, Jacqueline Stack, Jane Kohlhoff, Jennifer M. Jones, John Smoleniec, Ju-Lee Oei, Karen Sorensen, Laura Collie, Lynn Kemp, Meena Chandra, Paul Chay, Shanti Raman, Stephen Matthey, Sue Woolfenden, Trish Clark, Valsa Eapen and Victoria Blight
